# COVID-19 and Treatment and Immunization of Children—The Time to Redefine Pediatric Age Groups is Here

**DOI:** 10.5041/RMMJ.10433

**Published:** 2021-04-29

**Authors:** Klaus Rose, Jane M. Grant-Kels, Earl B. Ettienne, Oishi Tanjinatus, Pasquale Striano, David Neubauer

**Affiliations:** 1 klausrose Consulting, Riehen, Switzerland; 2 UConn Health, Farmington, CT, USA; 3 Howard University College of Pharmacy, Washington DC, USA; 4 Pediatric Neurology and Muscular Diseases Unit, Department of Neurosciences, Rehabilitation, Ophthalmology, Genetics, Maternal and Child Health, University of Genova, ‘G. Gaslini’ Institute, Genova, Italy; 5 Department of Child, Adolescent & Developmental Neurology, University Children’s Hospital, Ljubljana, Slovenia

**Keywords:** Better medicines for children, children as therapeutic orphans, COVID-19, developmental pharmacology, pediatric drug development, pediatric legislation

## Abstract

Children are infected with coronavirus disease 2019 (COVID-19) as often as adults, but with fewer symptoms. During the first wave of the COVID-19 pandemic, multisystem inflammatory syndrome (MIS) in children (MIS-C), with symptoms similar to Kawasaki syndrome, was described in young minors testing positive for COVID-19. The United States (US) Centers for Disease Control and Prevention (CDC) defined MIS-C as occurring in <21-year-olds, triggering hundreds of PubMed-listed papers. However, postpubertal adolescents are no longer children *biologically*; the term MIS-C is misleading. Furthermore, MIS also occurs in adults, termed MIS-A by the CDC. Acute and delayed inflammations can be triggered by COVID-19. The 18th birthday is an *administrative* not a *biological* age limit, whereas the body matures slowly during puberty. This blur in defining children leads to confusion regarding MIS-C/MIS-A. United States and European Union (EU) drug approval is handled separately for children, defined as <18-year-olds, ascribing non-existent physical characteristics up to the 18th birthday. This blur between the administrative and the physiological meanings for the term child is causing flawed demands for pediatric studies in all drugs and vaccines, including those against COVID-19. Effective treatment of all conditions, including COVID-19, should be based on actual physiological need. Now, the flawed definition for children in the development of drugs and vaccines and their approval is negatively impacting prevention and treatment of COVID-19 in minors. This review reveals the necessity for redefining pediatric age groups to rapidly establish recommendations for optimal prevention and treatment in minors.

## INTRODUCTION

The severe acute respiratory syndrome coronavirus 2 (SARS-CoV-2), which causes coronavirus disease 2019 (COVID-19), has led to havoc worldwide. The most important single prognostic factor is patient age.^1^,^2^ Initially, COVID-19 was thought not to affect children. However, it is now known that children are also easily infected, but most have no or few symptoms. Since most infected minors neither cough nor sneeze, their role as vectors in spreading the disease is limited. However, their role as a potential reservoir of the virus should be kept in mind.^3^,^4^

A multisystem inflammatory syndrome (MIS) in children (MIS-C) was described during the first wave of the COVID-19 pandemic,^5^,^6^ with some similarities to Kawasaki syndrome.^2^,^7^ Subsequently, the United States (US) Centers for Disease Control and Prevention (CDC) published a Health Advisory,^8^ defining MIS-C as occurring in patients <21 years old with fever, laboratory evidence of inflammation, illness requiring hospitalization, multisystem (≥2 organs) involvement, no alternative plausible diagnoses, and current or recent COVID-19 infection or COVID-19 exposure. The American Academy of Pediatrics (AAP) issued an interim guidance, listing potential complications and manifestations including Kawasaki disease-like features, toxic shock syndrome-like features, cytokine storm/macrophage activation or hyperinflammatory features, and more.^9^

The CDC health advisory triggered many PubMed-listed papers. As of January 1, 2021, the search terms “multisystem inflammatory syndrome COVID children” revealed 391 publications; the search terms “multisystem inflammatory syndrome COVID” 434 publications; and the search term “MIS-C” 204 publications.

Is the term MIS-C appropriate? The CDC health advisory^8^ included four references: a CDC description of Kawasaki disease^7^ plus three published papers.^5^,^6^,^10^ Kawasaki disease primarily affects children <5 years old.^7^ The Royal College of Paediatrics and Child Health (RCPCH) and Riphagen et al. provide guidance on diagnosis and treatment.^5^,^10^ Verdoni et al. had reported an increased number of children in an Italian pediatric hospital.^6^ A UK national consensus paper advised how to manage MIS-C.^11^

The first MIS-C reports *assumed* a pediatric challenge,^5^,^6^,^10^ and both the US CDC and the AAP defined MIS-C as occurring in patients <21 years old.^8^,^9^ Furthermore, the AAP defines the pediatric population as ≤21 years old, and even older for those with special needs.^12^ Although this is reasonable for *administrative* purposes, in reality 15- or 20-year-olds are *physically* no longer children. The UK authors do not define children.^10^,^11^ For them, a child would appear to be somebody seen by a pediatrician, which in our view reflects a circular and non-scientific perspective.

The term “child” has different meanings depending on the used context. Administratively and legally, a child is a minor. However, the body matures before administrative adulthood.^13^ In the past, minors became adults through religious and other traditional rites that ceremoniously continue today in some groups. Today, a child becomes an adult by passing an age limit. This age limit has been applied to scientific studies despite the fact that onset and completion of puberty has been noted to be accelerated in the last decades.^14^

Adults have been reported to experience MIS as a result of COVID-19 infection. In adults it is now referred to as MIS-A.^15^–^17^ This begs the question: If MIS can occur in both children and adults, is it appropriate to differentiate between MIS-C and MIS-A? Furthermore, if MIS-C and MIS-A have the same symptoms, is there a justification for separate clinical trials in MIS-C?

This question is not theoretical, but has practical implications. For example, one study compared the antibodies of COVID-19 patients with those of MIS-C patients, claiming they were different.^18^ The authors compared three groups: Group 1, recovered patients after mild COVID-19 respiratory disease; Group 2, hospitalized severe COVID-19 patients; and Group 3, children hospitalized with MIS-C. The ages of the groups are presented in Table 1. Note the overlapping age ranges for Groups 2 and 3; however, the upper age limit of Group 3 was the age of patients administratively allowed on a pediatric ward. Obviously, some adolescents in Group 2 were treated in adult wards. Weisberg et al. and most others use MIS-C uncritically.^18^

**Table 1:** Summary of the Age Groups Studied by Weisberg et al.^18^

Group	Median Age (Range) in Years
Group 1: Recovered patients after mild COVID-19 respiratory disease	45 (28–69)
Group 2: Hospitalized severe COVID-19 patients	56 (14–84)
Group 3: Children hospitalized with MIS-C	11 (4–17)

Note the overlapping age ranges for Groups 2 and 3.

Viral infections can induce various autoimmune disorders, including secondary hemophagocytic lymphohistiocytosis and macrophage activation syndrome.^16^,^19^,^20^ Multisystem inflammatory syndrome might be a macrophage activation syndrome.^16^,^20^ The immune responses to MIS may be related to a direct infection, an uncontrolled autoimmune response, or both.^19^

Many MIS-C patients were and are *physiologically* no longer children. Hence, there should be no differentiation between MIS-C and MIS-A: the term should be MIS only. This paper discusses the implications of differentiating between pediatric and adult designations with a focus first on COVID-19 and then on drug development and drug approval in general.

## REGULATORY HISTORY OF DRUG DEVELOPMENT

Most COVID-19 and MIS-C papers share the presentation and course of their patients and advise on treatment. This was how pediatrics and all clinical disciplines historically emerged and evolved: trying to help by whatever was at hand.^21^ For example, during the thalidomide disaster pregnant women took thalidomide for nausea and morning sickness; this led to serious defects to their child, with the most common defect being malformations of arms and legs (phocomelia). Reacting to this, in 1962 the US introduced the requirement to prove the effectiveness and safety of drugs before approval, a principle eventually accepted worldwide.^22^ The cornerstone of this proof is clinical studies. A somewhat later learning was that young persons differ from adults. Pediatricians had used formulas and tables for dosing, which usually worked fine. However, at very young ages, absorption, distribution, metabolism, and excretion are very different; formulas and tables could recommend doses that were too high or too low.^23^,^24^ As a protection against damaging lawsuits in the litigious US, companies began adding pediatric warnings to drug labeling as of 1962. The first chairman of the AAP committee on drugs claimed this disadvantaged children in drug treatment, making them “therapeutic orphans.”^25^ The AAP and the US Food and Drug Administration (FDA) took up this concept. In 1979, the FDA defined “children” as <17 years old.^26^ Since 1997, US law offers voluntary financial rewards for companies performing pediatric studies. Pediatric legislations became permanent in the US in 2012. The EU has been demanding, since 2007, that all new drugs that might later be used in minors have a pediatric investigation plan (PIP), including biologics, vaccines, and drugs that target rare diseases.^24^,^26^ Today, both the FDA and the European Medicines Agency (EMA) use the 18th birthday to distinguish adults from “children.”

The discussion about on-label versus off-label treatment is old, broad, and keeps US courts busy. Modern labeling has its merits. There are also good arguments against banning scientific discussion of off-label use. Once approved, drugs may be prescribed for any purpose. A physician’s failure to appropriately prescribe off-label would inhibit good care and could be considered potential medical malpractice.^27^ Imposing a separate pediatric approval had a negative impact on reasonable on-label/off-label use in the administratively labeled pediatric population. It is certainly justified in preterm newborns, but not in all children.

The demand for separate testing of drugs in children appears noble at first. Nonetheless, there is a catch: the semantic blur between the different meanings of the term “child.” Minors become bodily mature long before the 18th birthday.^23^ The children-are-therapeutic-orphans concept turns the real dangers of effective drugs in preterm newborns into alleged dangers for all drugs in all “children.” Relevant aspects of pediatric research have been moved from studies with therapeutic intent to studies aiming at acquiring pediatric labeling.^24^,^28^ Yet historically, the entire pediatric discipline developed “off-label” decades before this term emerged in 1988.^29^

As a result, the term MIS-C has been flawed from the beginning. The official CDC warning^8^ resulted in its worldwide adoption, triggering several hundred publications that further accentuate this weakness of modern medicine. Physicians are good at caring for patients within the existing framework. Hence, if an authority uses the term MIS-C, that term is generally accepted. The COVID-19 pandemic reveals how a flawed term is accepted, repeated worldwide, ultimately impacting the effectiveness of patient care.

## REGULATORY REQUIREMENTS FOR POTENTIAL VACCINES AND TREATMENTS OF COVID-19

The FDA and the EMA jointly published a document explaining their pediatric requirements for companies that develop vaccines or treatments against COVID-19.^30^ The EMA demands PIPs for virtually all new drugs, vaccines, and biologics. Up until 2017, the FDA could not mandate pediatric studies for drugs targeting a rare disease. However, since 2020, it requires pediatric studies for new anticancer drugs in patients ≤11 years old.^31^ The FDA also demands that companies submit an initial pediatric study plan (iPSP).^24^,^30^

### COVID-19 Treatment

During the COVID-19 pandemic, the FDA authorized remdesivir for emergency treatment of patients ≥12 years old, weighing ≥40 kg.^32^,^33^ The EMA issued a conditional marketing authorization for the same patient group.^34^ In October 2020, the FDA expanded this emergency authorization for hospitalized children weighing ≥3.5 kg, giving dosing recommendations based on the patient’s body weight.^35^ The pediatric studies required by the remdesivir PIP are listed in Box 1.^36^

Box 1 **Pediatric Studies Required by the EMA in the Remdesivir COVID-19 PIP**

1. Open-label single-arm pharmacokinetics, tolerability, and efficacy study in hospitalized children from 32 weeks’ gestational age to 17 years with confirmed COVID-19
2. Population pharmacokinetics modeling and simulation study on pediatric dose/posology in pediatric subjects 32 weeks’ gestational age to 17 years to achieve systemic exposures equivalent to those in adults
3. Extrapolation study on efficacy and safety of remdesivir from adults to children 32 weeks’ gestational age to 17 years with confirmed COVID-19

EMA, European Medicines Agency; PIP, pediatric investigation plan.

There is no known age limit that would require a fundamentally different treatment of young COVID-19 patients compared to the treatment of adults. Antivirals, monoclonal antibodies, and receptor blockers work the same before and after a person’s 18th birthday. For pre-adolescent children, dosing recommendations are needed, such as the ones issued by the FDA.^35^ However, all PIP-required pediatric COVID-19 studies follow the same rigid PIP scheme used by the EMA in all clinical areas.^24^ Furthermore, treating a MIS patient with remdesivir is medically contraindicated, since there is no longer direct inflammation by the virus; rather, the immune system is overreacting.^2^

### COVID-19 Vaccines

The FDA emergency approval of the first vaccine (BNT162b2) was for patients ≥16 years old,^37^ the second (Moderna) for those ≥18 years old.^38^ More vaccines are under development. In general, vaccines have helped to contain most viral infectious diseases, although there is no effective vaccine yet against human immunodeficiency virus. Clearly, minors will eventually need to be vaccinated against COVID-19. There is no scientifically valid reason to limit the age for administering the first two approved COVID-19 vaccines. A 15-year-old will not react differently from an 18-year-old. The decision to define the lower age limit of both pivotal vaccination studies was made by the developers.

Box 2 shows the pediatric studies demanded in the EMA PIP for BNT162b2.^39^

Box 2 **Pediatric Studies Demanded in the EMA SARS-CoV-2 Spike Protein (BNT162b2) PIP (EMEA-002861-PIP02-20)**

1. Double-blind dose-finding study of safety, tolerability, and immunogenicity of two different SARS-CoV-2 vaccine candidates (adults only) (part 1) and placebo-controlled efficacy, safety, and immunogenicity study of BNT162b2 in adolescents 12–17 years of age (and adults) (part 2) for prevention of COVID-19
2. Double-blind, controlled, dose-finding study of safety and immunogenicity of BNT162b2 in children and adolescents from 5–17 years of age for prevention of COVID-19
3. Double-blind, controlled, dose-finding, study of safety and immunogenicity of BNT162b2 in children from birth to <5 years of age for prevention of COVID-19
4. Open-label, uncontrolled study of safety and immunogenicity in immunocompromised children from birth to 17 years of age for prevention of COVID-19

EMA, European Medicines Agency; PIP, pediatric investigation plan; SARS-CoV-2, severe acute respiratory syndrome coronavirus 2.

All these studies were demanded in children aged from birth to 17 years. However, while 15-year-olds are *administratively* still children, their bodies are not. Once a compound is known to be effective in humans, double-blind placebo-controlled studies (points 2 and 3 in Box 2) are not necessary and therefore unethical. Dose-finding studies do not need to be double-blinded. The PIP-demanded studies (Box 2) lack medical necessity and represent a waste of money and time.

### Justifications for Separate Pediatric Drug Development

The FDA/EMA-employed authors and their allies in academic pediatric research and industry speak of therapeutic tragedies in pediatric patients,^40^ drug development in pediatric patients, 41,42 or developing medicines for children,^43^ assuming that all children of all ages are in danger of toxicities when treated with drugs. Such dangers exist in preterm newborns, and to a lesser degree in mature newborns, but clearly not in all children—particularly if “children” are defined as being up to the age of 18 or 21.

### Accelerated Pediatric Procedures

The question as to whether or not PIPs or iPSPs make sense for the COVID-19 pandemic was not even addressed by the FDA or the EMA. Instead, the need for pediatric drug development was assumed. However, the EMA emphasizes that it offers accelerated procedures for PIPs and related issues for COVID-19 treatments and vaccines.^44^ While the usual procedure to negotiate a PIP takes about a year,^24^ this procedure is accelerated for drugs and vaccines against COVID-19. Also, the FDA will not approve drugs and vaccines for COVID-19 and other diseases without separate “pediatric” data.

### Clinical Studies for COVID-19 Vaccines and Therapies

Table 2 shows industry-sponsored clinical studies for prevention and treatment of COVID-19. Most include patients ≥12 years old, some accept patients ≥6 years old, a few accept all ages, others ages 6–90 (#20) or 12–100 years (#17).

**Table 2:** **Industry-Sponsored COVID-19 Drug and Vaccine National Clinical Trials (NCT) Found in** www.clinicaltrials.gov **Using Search Terms: COVID-19; Pediatric; Industry-sponsored**.

#	NCT No.	Abbreviated Study Design	Age (Number; Centers*)	Status
1	04551547	R, DB, PC vaccine study	3–17y (*n*=552)	Recruiting
2	04456439	EAP of HMSC for MIS-C	2mo–17y (*n*=~50)	Available
3	04649151	PC mRNA-1273 Vaccine S, RG, E study	12–17y (*n*=3,000; 7 ctrs)	Not yet recruiting
4	04431453	S, T, PK, E of remdesivir	<17y (*n*=52; 30 ctrs)	Recruiting
5	04646031	Open label EAP of T89 botanical drug	C+A+OA	Available
6	04368728	R, PC, BNT162b1 + BNT162b2	>12y (*n*=43,998; 155 ctrs)	Recruiting
7	04362137	R, DB, PC ruxolitinib in CS	>12y (*n*=432; 61 ctrs)	Completed
8	04409262	R+T versus R+P in severe pneumonia	>12y (*n*=500; 73 ctrs)	Recruiting
9	04597047	MC diagnostic study	C+A+OA (*n*=200)	Recruiting
10	04355793	EAP ruxolitinib in CS	>12y	Recruiting
11	04377620	PC ruxolitinib in ventilated ARDS pts	>12y (*n*=500; 35 ctrs)	Recruiting
12	04362189	PC study of HB-adMSCs	C+A+OA (*n*=100)	Active non-recruiting
13	04486313	PC, S, E of nitazoxanide	>12y (*n*=800)	Recruiting
14	04427501	PC, R, LY-CoV555 + LY-CoV016	>12y (*n*=1,200)	Recruiting
15	04292899	S, E of remdesivir in severe COVID-19	>12y (*n*=4,891; 183 ctrs)	Completed
16	04501952	S, E of remdesivir in outpatients	>12y (*n*=1,264; 45 ctrs)	Recruiting
17	04377711	R, DB, PC ciclesonide in outpatients	12–100y (*n*=400)	Recruiting
18	04348435	R, DB, PC, S, E, HB-adMSCs to protect against COVID-19	C+A+OA (*n*=100)	Enrolling by invitation
19	04292730	Remdesivir vs. SoC	>12y (*n*=1,113; 184 pts)	Completed
20	04337359	Ruxolitinib MAP	6–90y	Available
21	04362813	DB, PC, S, E canakinumab in pneumonia	>12y (*n*=451)	Active non-recruiting
22	04422509	Lanadelumab	>16y (*n*=80; 10 ctrs)	Recruiting
23	04566770	Vaccine study	>6y (*n*=481)	Recruiting
24	04471519	DB, PC, vaccine study	12–65y (*n*=755)	Recruiting
25	04617535	Compassionate use REGN-COV2	C+A+OA	Available
26	04603651	Bamlanivimab EAP	>12y	No longer available
27	04349631	HB-adMSCs to protect against COVID-19	C+A+OA (*n*=56)	Active non-recruiting
28	04323761	EAP remdesivir	>12y	Approved for marketing
29	04360096	Inhaled aviptadil	12–85y (*n*=288)	Not yet recruiting
30	04453839	Aviptadil EAP	12–100y (*n*=24)	Available
31	03808922	PC, DAS181 (1 subgroup only)	C+A+OA (*n*=274; 58 ctrs)	Recruiting
32	04452318	PC, REGN10933+REGN10987 protection	>12y (*n*=2,000)	Recruiting
33	04579640	Open-label randomized vitamin D to reduce risk and severity of COVID-19	>16y (*n*=6,200)	Active non-recruiting

* Number relates to patients or participants, as defined in the study; not all studies provided both number of patients and number of centers.

ARDS, acute respiratory distress syndrome; C+A+OA, child+adult+older adult; CS, cytokine storm; DB, double-blind; E, efficacy; EAP, expanded access program; HB-adMSCs, Hope Biosciences allogeneic adipose-derived mesenchymal stem cells; HMSC, human mesenchymal stromal cells; MAP, managed access program; MC, multicenter; MIS-C, multisystem inflammatory syndrome in children; mo, months; PC, placebo-controlled; PK, pharmacokinetics; R, remdesivir; R+P, remdesivir+placebo; R+T, remdesivir+tocilizumab; RG, reactogenicity; S, safety; SoC, standard of care; T, tolerability; y, years.

Table 3 lists the ongoing clinical studies for MIS-C related to COVID-19 that were found in a PubMed search. Most of these studies are observational or diagnostic, but #1 and #5 offer treatment with mesenchymal stroma cells; #6 plans for “children” aged 18–20 years; and #1 is for “children” ranging from 2 months to 17 years. Again, these inclusion criteria are neither based on biological/physical definitions, nor are they consistent.

**Table 3:** MIS-C National Clinical Trials (NCT) Listed in PubMed.

#	NCT No.	Abbreviated Study Description	Age	Patients, *n*	Status
1	04456439	Intermediate-size EAP (MSC) for MIS-C associated with COVID-19	2mo–17y	~50	Available
2	04588363	OS in COVID-19 infections and MIS-C	<20y	250	Recruiting
3	04538495	OS: characterization of MIS-C and its relationship to Kawasaki Disease	>1mo	100	Recruiting
4	04640038	Contrast-enhanced ultrasound in COVID-19	<17y	30	Recruiting
5	04659486	Adolescents with COVID-19/MIS-C at HCFMUSP	7–18y	100	Enrolling by invitation
6	04549285	MSC infusions in children with MIS-C	18–20y	6	Not yet recruiting

EAP, expanded access program; HCFMUSP, Hospital das Clínicas da Faculdade de Medicina da USP, Sao Paolo, Brazil; mo, month; MIS-C, multisystem inflammatory syndrome in children; MSC, mesenchymal stromal cells; OS, observational study; y, year.

## DISCUSSION

The terms MIS-C and MIS-A mirror the artificial division of humans into two populations: adults and children. For legal and/or administrative purposes, classifications into age groups are justified. However, when treating patients, the diagnoses should be the same for identical conditions/syndromes presenting with the same symptoms and diagnostic tests, with the same treatments being offered. A diagnosis should not be one thing the day before a specific birthday, and something different the day after—nor should the treatments differ.

From the beginning, the AAP guidelines on pediatric studies have oscillated between the legal and the physiological meanings of the term “child.”^45^,^46^ As stated in the introduction, the AAP defines the pediatric population as ≤21 years old, and even allows for a higher age limit for patients with special needs.^12^ This is acceptable for administrative purposes. The flawed FDA definition of a pediatric population as <17 years originated in 1979.^47^ Pediatric drug development^39^ was established in a first US law in 1997, which rewarded separate pediatric studies by patent extension.^24^,^26^ This was expanded upon by the EU, which has demanded PIPs for new drugs, vaccines, and biologics that might later be used in minors since 2007. Today, both the FDA and the EMA use the age limit of 18 years.^24^,^48^

The semantic confusion regarding MIS-C/MIS-A has a dark side. Since 1974, the AAP has justified separate pediatric studies, characterizing them as a “moral imperative.”^43^,^44^ The FDA’s demands for questionable pediatric cancer studies in patients ≤21 years old should be seen in this context.^48^–^51^ Once puberty is completed, the body is mature.^13^,^14^

Therefore, dosing recommendations should be based on modeling and simulation calculations, confirmed in an opportunistic framework not requiring complex logistics, and without exposing patients to separate exaggerated studies.^52^,^53^ In this context, the term “opportunistic” describes the opportunity to gather additional data when a minor needs to be treated with an innovative drug that is not yet formally approved in this age group.

Another disease that exemplifies this issue is juvenile idiopathic arthritis (JIA), a name that suggests juvenile patients. *Historically*, arthritides in young patients were first investigated by pediatric rheumatologists.^54^ First introduced in 1995, JIA was an umbrella term for diseases that began before the 16th birthday.^55^ Before 1995, the terms juvenile rheumatoid arthritis and juvenile chronic arthritis were used. Notably, several diseases under today’s JIA umbrella affect adults under different names: adult-onset Still’s disease (AOSD) and systemic JIA (sJIA) are the same, as are rheumatoid factor-positive (RF+) polyarthritis (a JIA category) and RF+ rheumatoid arthritis; and enthesitis-related arthritis (another JIA category) and adult undifferentiated spondyloarthritis (SpA).^56^,^57^ Investigations for arthritides in young patients began when infectious diseases had been pushed back and a focus on rare diseases in young patients emerged, including cancer and arthritis in minors.^24^,^50^,^51^ However, diseases do not change at the 16th or 18th birthday.^58^ Today there is a “Center for Adults with Pediatric Rheumatic Illness (CAPRI).”^59^

The basic assumption behind pediatric drug development is that children and their diseases are fundamentally different from adults,^60^ requiring separate pediatric studies. Today, we know how the fetus develops in the mother,^61^ and how children grow from birth into adulthood.^13^,^14^,^23^ While the APP and other organizations recognize the physiological differences between infants, “children,” and “adolescents,” the administrative age limit across the board remains the determining factor for all “pediatric” studies.

At the interface of drug development and drug approval a coalition has emerged that refuses to apply the learnings of developmental pharmacology. Instead, it demands separate pediatric studies. This coalition is composed of academic researchers, regulatory authorities, pharmaceutical companies, and clinical trials organizations. This requirement has evolved into a business opportunity providing funds for pediatric research.^24^,^48^,^50^

Some clinicians have challenged this situation.^62^,^63^ In epilepsy, the FDA today accepts “extrapolation” of efficacy from adults down to 2 years of age.^64^ But in this context, the term “extrapolation” is flawed. Dosing needs extrapolation; efficacy does not. Antiepileptic drugs work in both adults and children.^65^

Other clinical areas still use “pediatric,” e.g. the International Pediatric Multiple Sclerosis Study Group (IPMSSG).^66^ While the term “pediatric” is incorrect for adolescents, the IPMSSG speaks out against “pediatric” placebo-controlled studies.^24^,^67^

Most regulatory “pediatric” studies lack medical sense. Many cause harm by withholding effective treatment or by exposing patients to treatment below standard-of-care.^24^,^48^,^49^,^62^ Together, regulatory pediatric studies amount to abuse in medical research that numerically exceeds the abuses reported by Beecher,^68^ and the Tuskegee study.^24^,^69^,^70^

The FDA is gradually stepping back from demanding separate pediatric studies,^24^,^46^,^49^,^62^ but the EMA is expanding on their demands, now requiring PIPs for drugs against liver carcinoma, kidney carcinoma, coronary atherosclerosis, Parkinson’s disease, Huntington’s chorea, and amyotrophic lateral sclerosis.^71^ These diseases occur occasionally before the 18th birthday, but are not, by any sense of the word, pediatric.

The FDA and the EMA maintain the children-are-therapeutic-orphans concept.^29^ For remdesivir pediatric dosing, the FDA has applied science,^34^ while the EMA is caught in a dogmatic labyrinth, demanding pointless pediatric studies (Tables 3 and 4).

Pediatric studies recruit worldwide, including in the US. Minors in the US will be threatened by EMA-mandated pediatric COVID-19 studies once these begin. As long as Institutional Review Boards (IRBs) and ethics committees (ECs) do not recognize the dangers of pediatric studies, the temptation for medical doctors and research institutions to participate in international pediatric studies will continue.

This is not the first time that mainstream science has supported a flawed concept. Sometimes, it takes decades or even centuries to change prevalent thinking.^72^

Off-label treatment will always be required when treating newly emerging diseases. The current COVID-19 pandemic will not be the world’s last pandemic.

## CONCLUSIONS

It is time for IRBs and ECs to recognize the danger of many pediatric studies, not only for patients, but also as they relate to public trust in medicine, research, and authorities. Pediatric research needs to return to the roots of medical care. What began as a flawed interpretation of legal warnings—and the resulting pediatric labeling—became institutionalized by US pediatric laws, was expanded upon by the EU, and is now maintained due to conflicts of interest. This situation will not disappear overnight.

Nevertheless, there are ways to get things started in the right direction. The COVID-19 pandemic presents an optimal opportunity for beginning. To start with, the CDC should retract and correct its MIS-C warning.^8^ Questionable pediatric studies should be suspended by IRBs/ECs, and newly submitted ones should be rejected. Journals should no longer accept the terms MIS-C and MIS-A in the titles or keywords of publications. Ultimately, US and EU pediatric laws need revision. Pediatricians and general practitioners should work with their professional organizations to achieve this goal. A concerted effort by healthcare practitioners who fight daily for their patients’ lives can have a powerful impact to achieve these goals.

## Abbreviations

CDC: Centers for Disease Control and Prevention
COVID-19: coronavirus disease 2019
EMA: European Medicines Agency
FDA: Food and Drug Administration
iPSP: initial pediatric study plan
JIA: juvenile idiopathic arthritis
MIS: multiple inflammatory syndrome
MIS-A: multiple inflammatory syndrome in adults
MIS-C: multiple inflammatory syndrome in children
PIP: pediatric investigation plan
SARS-CoV-2: severe acute respiratory syndrome coronavirus 2
US: United States.

## References

[1] Tabary M, Khanmohammadi S, Araghi F, Dadkhahfar S, Tavangar SM (2020). Pathologic features of COVID-19: a concise review. Pathol Res Pract.

[2] Jiang L, Tang K, Levin M (2020). COVID-19 and multisystem inflammatory syndrome in children and adolescents. Lancet Infect Dis.

[3] Zimmermann P, Curtis N (2020). Coronavirus infections in children including COVID-19: an overview of the epidemiology, clinical features, diagnosis, treatment and prevention options in children. Pediatr Infect Dis J.

[4] Naja M, Wedderburn L, Ciurtin C (2020). COVID-19 infection in children and adolescents. Br J Hosp Med (Lond).

[5] Riphagen S, Gomez X, Gonzalez-Martinez C, Wilkinson N, Theocharis P (2020). Hyperinflammatory shock in children during COVID-19 pandemic. Lancet.

[6] Verdoni L, Mazza A, Gervasoni A (2020). An outbreak of severe Kawasaki-like disease at the Italian epicentre of the SARS-CoV-2 epidemic: an observational cohort study. Lancet.

[7] US Department of Health and Human Services (2020). Kawasaki syndrome Centers for Disease Control and Prevention (CDC) website.

[8] US Department of Health and Human Services Emergency preparedness and response: HAN00432 (2020). Health Alert Network (HAN), Official CDC Health Advisory Centers for Disease Control and Prevention (CDC) website.

[9] American Academy of Pediatrics (AAP) (2020). Multisystem inflammatory syndrome in children (MIS-C) interim guidance. AAP website.

[10] Royal College of Paediatrics and Child Health (RCPCH) (2020). Paediatric multisystem-inflammatory syndrome temporally associated with COVID-19 (PIMS) – guidance for clinicians. RRCPCH website.

[11] Harwood R, Allin B, Jones CE (2021). A national consensus management pathway for paediatric inflammatory multisystem syndrome temporally associated with COVID-19 (PIMS-TS): results of a national Delphi process. Lancet Child Adolesc Health.

[12] Hardin AP, Hackell JM, Committee on Practice and Ambulatory Medicine (2017). Age limit of pediatrics. Pediatrics.

[13] Beunen GP, Rogol AD, Malina RM (2006). Indicators of biological maturation and secular changes in biological maturation. Food Nutr Bull.

[14] Komlos J, Lauderdale BE (2007). The mysterious trend in American heights in the 20th century. Ann Hum Biol.

[15] Morris SB, Schwartz NG, Patel P (2020). Case series of multisystem inflammatory syndrome in adults associated with SARS-CoV-2 infection - United Kingdom and United States, March-August 2020. MMWR Morb Mortal Wkly Rep.

[16] Theoharides TC, Conti P (2020). COVID-19 and multisystem inflammatory syndrome, or is it mast cell activation syndrome?. J Biol Regul Homeost Agents.

[17] US Department of Health and Human Services Multisystem inflammatory syndrome in adults (2020). Centers for Disease Control and Prevention (CDC) website.

[18] Weisberg SP, Connors T, Zhu Y (2020). Antibody responses to SARS-CoV2 are distinct in children with MIS-C compared to adults with COVID-19. medRxiv.

[19] Icenogle T (2020). COVID-19: infection or autoimmunity. Front Immunol.

[20] Giamarellos-Bourboulis EJ, Netea MG, Rovina N (2020). Complex immune dysregulation in COVID-19 patients with severe respiratory failure. Cell Host Microbe.

[21] Colón AR (1999). Nurturing Children A History of Pediatrics.

[22] Rägo L, Santo B, van Boxtel CJ, Santo B, Edwards IR (2008). Drug Regulation: History, Present and Future. Drug Benefits and Risks: International Textbook of Clinical Pharmacology Revised.

[23] Kearns GL, Abdel-Rahman SM, Alander SW, Blowey DL, Leeder JS, Kauffman RE (2003). Developmental pharmacology -- drug disposition, action, and therapy in infants and children. N Engl J Med.

[24] Rose K (2020). Considering the Patient in Pediatric Drug Development. How Good Intentions Turned into Harm.

[25] Shirkey H (1968). Therapeutic orphans. J Pediatr.

[26] Hirschfeld S, Saint-Raymond A (2011). Pediatric regulatory initiatives. Handb Exp Pharmacol.

[27] Janssen WM, Levy MC (2008). A Historical Perspective on Off-label Medicine: from Regulation, Promotion, and the First Amendment to the Next Frontiers. Off-label Communications.

[28] US Food & Drug Administration (2019). Pediatric Research Equity Act (PREA).

[29] Plate V The impact of off-label, compassionate and unlicensed use on health care laws in preselected countries. Bonn, Germany, 2009. Dissertation.

[30] US Food & Drug Administration, European Medicines Agency (2020). FDA /EMA common commentary on submitting an initial pediatric study plan (iPSP) and paediatric investigation plan (PIP) for the prevention and treatment of COVID-19.

[31] Reaman GH FDARA 2017 and the RACE for Children Act: Implications for pediatric cancer drug development.

[32] US Food & Drug Administration Highlights of Prescribing Information: VEKLURY® (remdesivir) prescribing information. https://www.accessdata.fda.gov/drugsatfda_docs/label/2020/214787Orig1s000lbl.pdf.

[33] Eastman RT, Roth JS, Brimacombe KR (2020). Remdesivir: A review of its discovery and development leading to emergency use authorization for treatment of COVID-19. ACS Cent Sci.

[34] European Medicines Agency – Science Medicines Health (2020). EMA/67677/2020. Veklury (remdesivir).

[35] FDA Fact Sheet for Healthcare Providers Emergency Use Authorization (EUA) of Veklury^®^ (Remdesivir) for Hospitalized Pediatric Patients Weighing 3.5 kg to Less Than 40 kg or Hospitalized Pediatric Patients Less Than 12 Years of Age Weighing at Least 3.5 kg.

[36] European Medicines Agency – Science Medicines Health (2020). EMA/269449/2020. European Medicines Agency decision, P/0201/2020.

[37] FDA (2020). Fact Sheet for Healthcare Providers Administering Vaccine (Vaccination Providers). Emergency Use Authorization (EUA) of the Pfizer-BioNTech COVID-19 Vaccine to Prevent Coronavirus Disease 2019 (COVID-19).

[38] ModernaTX, Inc (2020). Fact Sheet for Healthcare Providers Administering Vaccine (Vaccination Providers). Emergency Use Authorization (EUA) of the Moderna COVID-19 Vaccine to Prevent Coronavirus Disease 2019 (COVID-19).

[39] European Medicines Agency – Science Medicines Health (2020). EMA/640530/2020. European Medicines Agency decision, P/0480/2020.

[40] Ward RM, Benjamin DK, Davis JM (2018). The need for pediatric drug development. J Pediatr.

[41] Ollivier C, Mulugeta YL, Ruggieri L (2019). Paediatric extrapolation: a necessary paradigm shift. Br J Clin Pharmacol.

[42] Bucci-Rechtsweg C (2017). Enhancing the pediatric drug development framework to deliver better pediatric therapies tomorrow. Clin Ther.

[43] van Riet-Nales DA, Römkens EG, Saint-Raymond A (2014). Oral medicines for children in the European paediatric investigation plans. PLoS One.

[44] European Medicines Agency (2020). EMA initiatives for acceleration of development support and evaluation procedures for COVID-19 treatments and vaccines.

[45] American Academy of Pediatrics. Committee on Drugs (1977). Guidelines for the ethical conduct of studies to evaluate drugs in pediatric populations. Pediatrics.

[46] Committee on Drugs, American Academy of Pediatrics (1995). Guidelines for the ethical conduct of studies to evaluate drugs in pediatric populations. Pediatrics.

[47] Hirschfeld S History of Pediatric Labeling [PowerPoint presentation]. https://www.slideserve.com/marlin/history-of-pediatric-labeling.

[48] Rose K, Tanjinatus O, Grant-Kels JM, Ettienne EB, Striano P, Neubauer D (2020). Minors and a dawning paradigm shift in “pediatric” drug development. J Clin Pharmacol.

[49] Reaman G (2014). Ipilimumab written request letter.

[50] Rose K, Grant-Kels JM (2018). Pediatric melanoma – the whole (conflicts of interest) story. Int J Womens Dermatol.

[51] Rose K (2020). Pediatric oncology at the crossroads: a call for change. Pharmaceut Med.

[52] Korth-Bradley JM (2018). The path to perfect pediatric posology - drug development in pediatrics. Clin Pharmacol.

[53] Gonzalez D, Melloni C, Yogev R (2014). Use of opportunistic clinical data and a population pharmacokinetic model to support dosing of clindamycin for premature infants to adolescents. Clin Pharmacol Ther.

[54] Brunner HI, Rider LG, Kingsbury DJ (2018). Pediatric Rheumatology Collaborative Study Group - over four decades of pivotal clinical drug research in pediatric rheumatology. Pediatr Rheumatol Online J.

[55] International League of Associations for Rheumatology (ILAR) Official organization website.

[56] Nigrovic PA, Raychaudhuri S, Thompson SD (2018). Review: Genetics and the classification of arthritis in adults and children. Arthritis Rheumatol.

[57] Martini A, Ravelli A, Avcin T (2019). Toward new classification criteria for juvenile idiopathic arthritis: first steps, pediatric rheumatology international trials organization international consensus. J Rheumatol.

[58] Rose K, Tanjinatus O, Ettienne E (2021). The term “juvenile idiopathic arthritis (JIA)” is misleading. It will not be sufficient to just replace this term. Pharmaceut Med.

[59] Brigham Health: Brigham and Women’s Hospital (2021). Pediatric Rheumatology for Adults.

[60] Wharton GT, Murphy MD, Avant D (2014). Impact of pediatric exclusivity on drug labeling and demonstrations of efficacy. Pediatrics.

[61] Gasser RF, Cork RJ, Stillwell BJ, McWilliams DT (2014). Rebirth of human embryology. Dev Dyn.

[62] Pellock JM, Carman WJ, Thyagarajan V, Daniels T, Morris DL, D’Cruz O (2012). Efficacy of antiepileptic drugs in adults predicts efficacy in children: a systematic review. Neurology.

[63] Pellock JM, Arzimanoglou A, D’Cruz O (2017). Extrapolating evidence of antiepileptic drug efficacy in adults to children > 2 years of age with focal seizures: the case for disease similarity. Epilepsia.

[64] US Department of Health and Human Services, Food and Drug Administration (2019). Drugs for treatment of partial onset seizures: full extrapolation of efficacy from adults to pediatric patients 2 years of age and older guidance for industry.

[65] Rose K, Neubauer D, Grant-Kels JM (2020). Ethical issues in pediatric regulatory studies involving placebo treatment. J Pediatr Epilepsy.

[66] International Pediatric Multiple Sclerosis Study Group (PPMSSG) Official website.

[67] Waubant E, Banwell B, Wassmer E (2019). Clinical trials of disease-modifying agents in pediatric MS: opportunities, challenges, and recommendations from the IPMSSG. Neurology.

[68] Beecher HK (1966). Ethics and clinical research. N Engl J Med.

[69] Adashi EY, Walters LB, Menikoff JA (2018). The Belmont Report at 40: reckoning with time. Am J Public Health.

[70] Rose CD (2017). Ethical conduct of research in children: pediatricians and their IRB (Part 1 of 2). Pediatrics.

[71] European Medicines Agency (2015). EMA/PDCO Summary report on the review of the list of granted Class Waivers.

[72] Zanatta A, Zampieri F, Basso C, Thiene G (2017). Galileo Galilei: science vs. faith. Glob Cardiol Sci Pract.

